# Supplementary material for: The deubiquitinase MYSM1 dampens NOD2-mediated inflammation and tissue damage by inactivating the RIP2 complex
Source: Nat Commun. 2018 Nov 7;9:4654. doi: 10.1038/s41467-018-07016-0 (PMC6220254; doi:10.1038/s41467-018-07016-0)
Supplement: Supplementary file 1 — Supplementary Information [file 41467_2018_7016_MOESM1_ESM.pdf]

Supplementary Information for

The deubiquitinase MYSM1 dampens NOD2-mediated inflammation and tissue damage by inactivating the RIP2 complex

Panda S and Gekara N.O

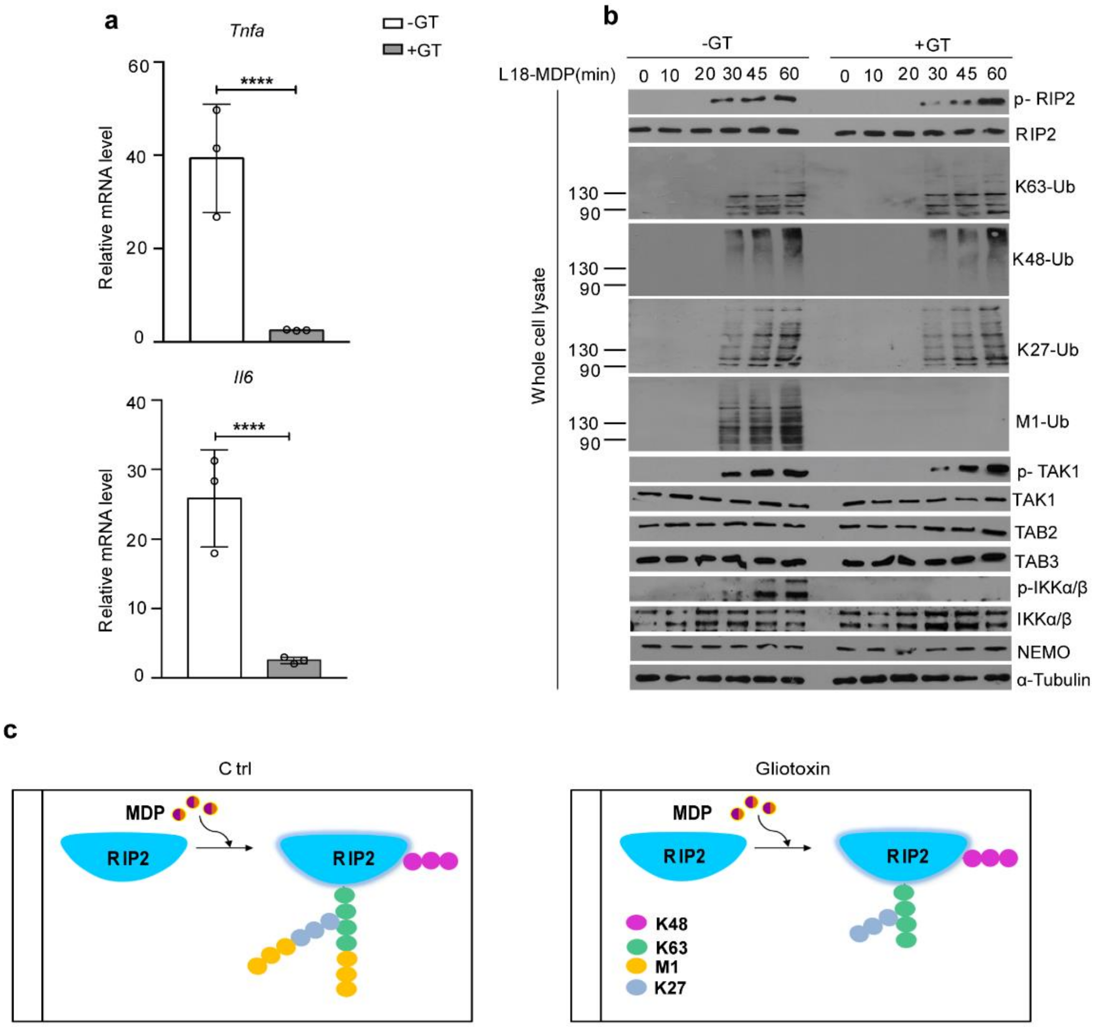

Supplementary Figure 1. Related to Figure 1. Inhibition of LUBAC blocks M1 but not K63, K48 or K27 polyubiquitination of RIP2. (**a**) WT BMDMs stimulated with L18-MDP for 6 h both in the presence (+) or absence (-) of gliotoxin (GT) were analyzed by qRT-PCR for *Tnfa* and *Il6* transcript. Results in **a** are from three independent experiments. Data are shown as mean ± s.e.m. (n=3). ***p < 0.001 determined by one-way ANOVA followed by Bonferroni's post-test depicts statistical significance relative to –GT. (**b**) Whole cell lysates from WT BMDMs stimulated with L18-MDP in the presence or absence of GT were analyzed for the indicated molecules (corresponds to Fig. 1d). Data are representative of three independent experiments. (**c**) Schematics: Gliotoxin blocks M1-, but not K63-, K48- and K27-linked polyubiquitination of RIP2.

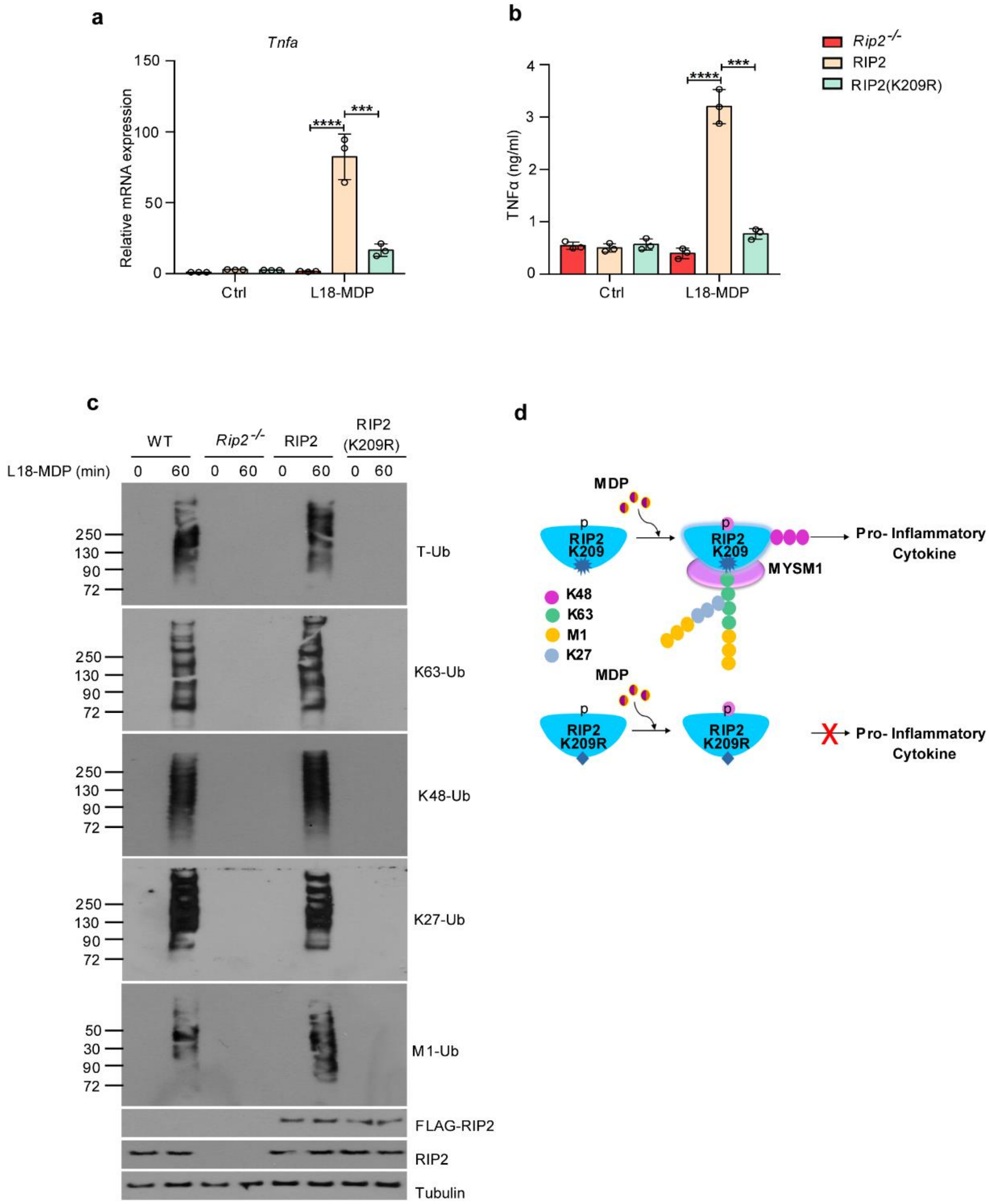

Supplementary Figure 2. RIP2 ubiquitination at Lysine 209 is crucial for NOD2 signaling. (**a-b**) *Rip2*$^{-/-}$ BMDMs complemented with full length RIP2 or RIP2(K209R) were stimulated with L18-MDP and then analyzed for *Tnfa* mRNA transcript 6 h later (**a**) or for secreted TNF-α after 12 h (**b**). Results in **a-b** are from three independent experiments. Data are shown as mean ± s.e.m. (n=3). ***p < 0.001 determined by one-way ANOVA followed by Bonferroni's post-test depicts statistical significance relative to RIP2. **(c)** WT and *Rip2*$^{-/-}$ BMDMs complemented (or not) with indicated RIP2 constructs were stimulated with L18-MDP and whole cell lysates were examined for the presence of indicated molecules. Data in **c** are representative of three independent experiments. (**d**) Schematics of MDP-induced RIP2 polyubiquitination and recruitment of MYSM1 to NOD2:RIP2 complex.

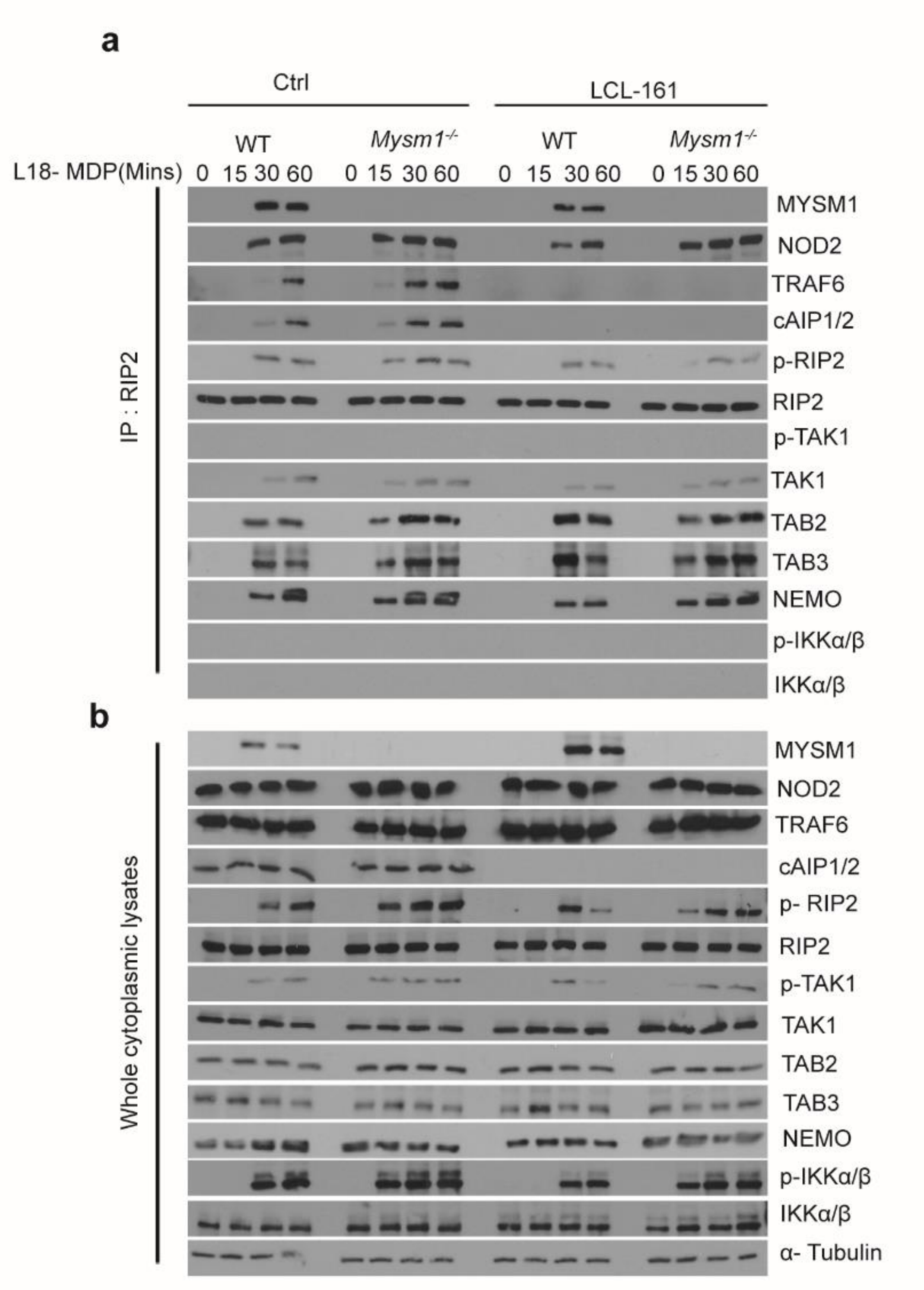

Supplementary Figure 3. MYSM1-mediated suppression of NOD2 signaling is independent of TRAF6 or cIAP1/2 proteins. (**a-b**) WT and *Mysm1*$^{-/-}$ BMDMs were stimulated (or not) with L18-MDP for the indicated duration in the presence or absence of the IAPs inhibitor LCL-161. Cytoplasmic fractions were immunoprecipitated with anti-RIP2. Pull downs (**a**) and the corresponding cytoplasmic fractions (**b**) were analysed for indicated molecules. Data are representative of three independent experiments.

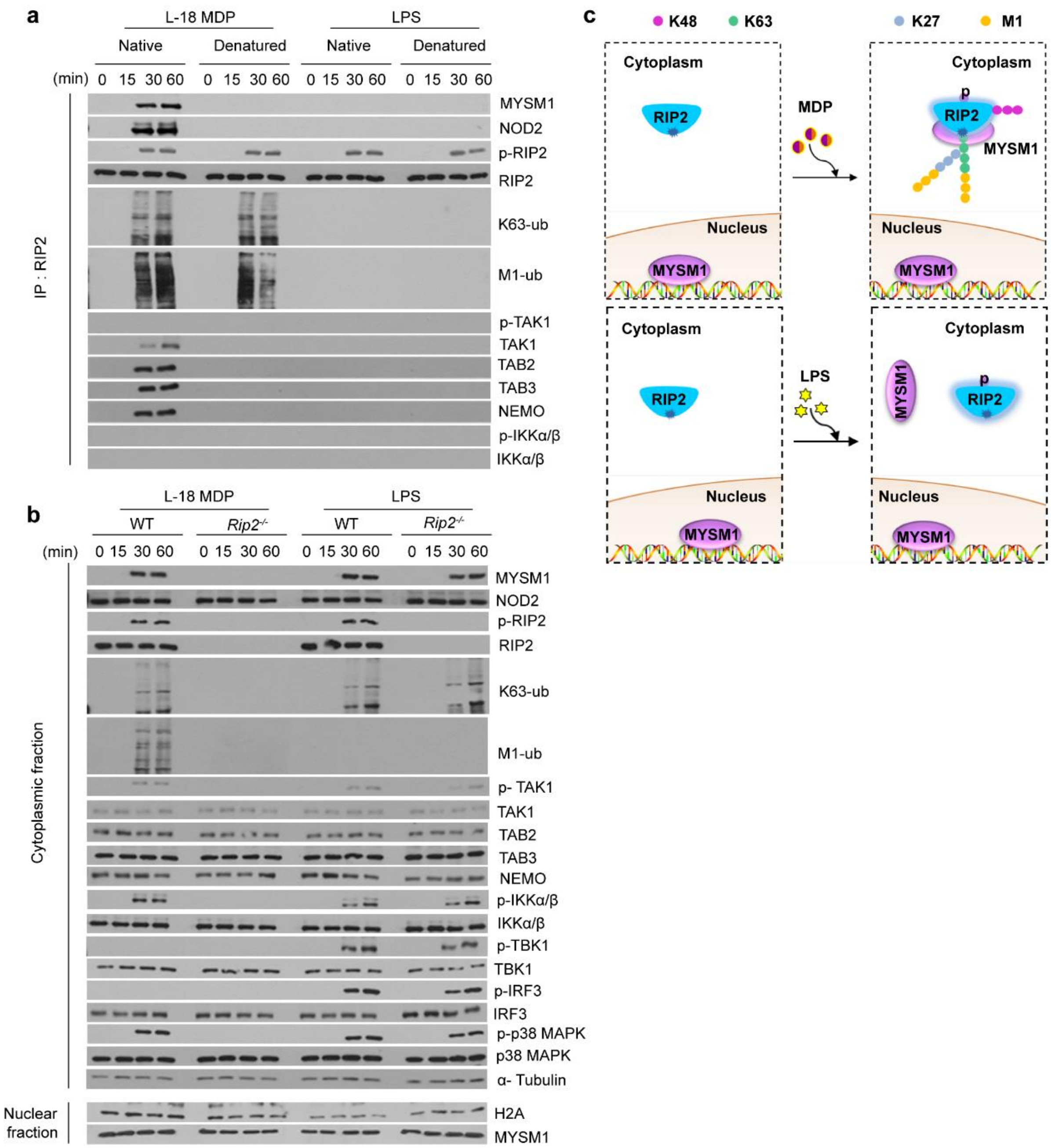

Supplementary Figure 4. Polyubiquitination of RIP2 is essential for the docking of MYSM1 to RIP2. (**a**) RIP2 was immunoprecipitated from native or denatured cytoplasmic fractions of L18-MDP- or LPS-stimulated WT BMDMs and then immunoblotted for indicated molecules. (**b**) Cytoplasmic and nuclear fractions from WT or *Rip2*$^{-/-}$ BMDMs were immunoblotted for indicated molecules. (**c**) Schematics: Triggering of NOD2, but not TLR4, induces RIP2 polyubiquitination and MYSM1 recruitment. Data are representative of three independent experiments.

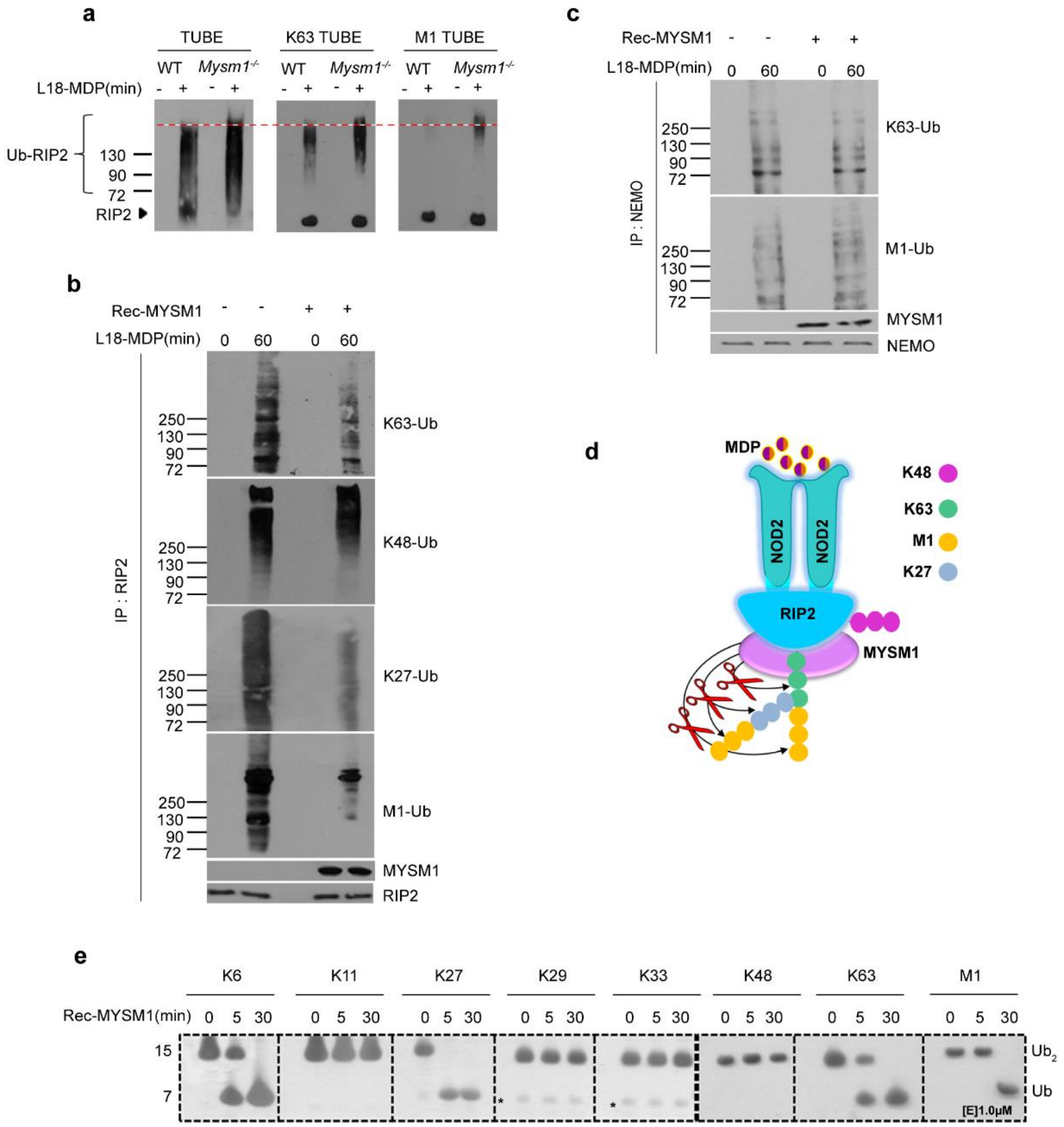

Supplementary Figure 5. Related to Figure 5. MYSM1 removes K63-, K27-, M1- but not K48-linked ubiquitin chains from RIP2, but not from NEMO. (**a**) Total, K63 and M1-linked polyubiquitinated proteins, isolated by TUBE, K63-TUBE and M1-TUBE, respectively, from WT and *Mysm1*$^{-/-}$ BMDMs stimulated with L18-MDP (1 h), were immunoblotted for RIP2. The dotted red line is an arbitrary line highlighting the presence of higher MW Ub-RIP2 in *Mysm1*$^{-/-}$ BMDMs. (**b**, **c**) *Mysm1*$^{-/-}$ BMDMs stimulated (or not) with L18-MDP for 1 h were immunoprecipitated with anti-RIP2 (**b**) or NEMO (**c**). Pull downs were incubated with recombinant (Rec)-MYSM1 and then immunoblotted with antibodies against indicated ubiquitin linkages. (**d**) Schematics: MYSM1-mediates cleavage of K63-, K27-, M1-, but not K48-linked polyubiquitinatin chains from RIP2. (**e**) Ubiquitin linkage specificity of MYSM1. Di-Ubiquitins of K6, K11, K27, K29, K33, K48, K63 and M1 linkages were incubated with Rec-MYSM1. Digestions were separated by SDS-PAGE and visualized by silver staining; (*****) indicates impurities of monoubiquitins. Data are representative of three independent experiments.

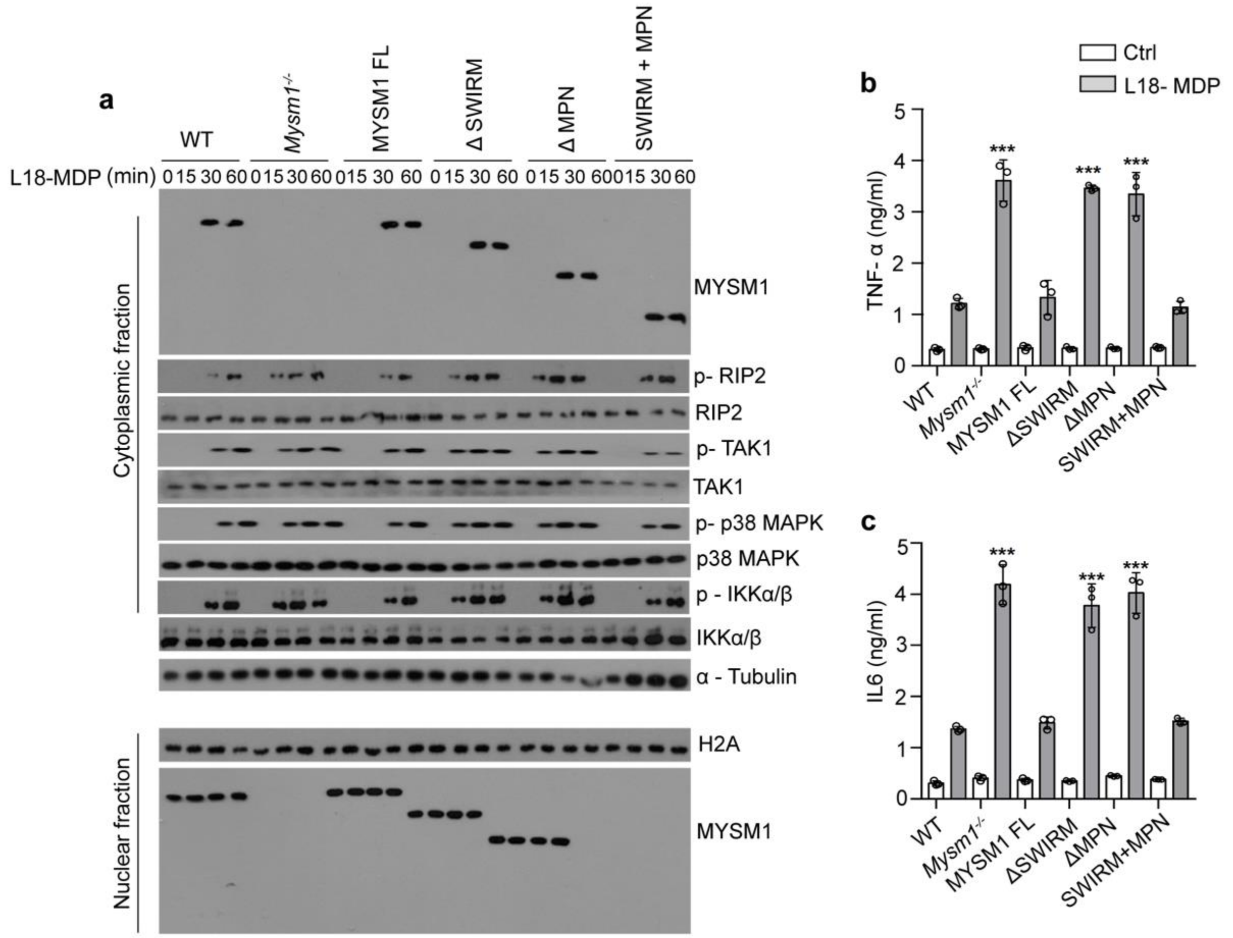

Supplementary Figure 6. Related to Figure 6. MYSM1 interacts with and inactivates RIP2 complex via the SWIRM and MPN domains. (a) WT, *Mysm1*$^{-/-}$ BMDMs or *Mysm1*$^{-/-}$ BMDMs complemented with indicated MYSM1 constructs were stimulated with L18-MDP and the corresponding cytoplasmic or nuclear fractions were analyzed for indicated molecules. Data are representative of three independent experiments. (**b-c**) Above-mentioned cells were stimulated with L18-MDP for 12 h and analysed for TNF-α (**b**) and IL-6 (**c**) secretion. Results in **b-c** are from three independent experiments. Data are shown as mean ± s.e.m., (n=3). ***$p < 0.001$ determined by one-way ANOVA followed by Bonferroni's post-test depicts statistical significance relative to Ctrl.

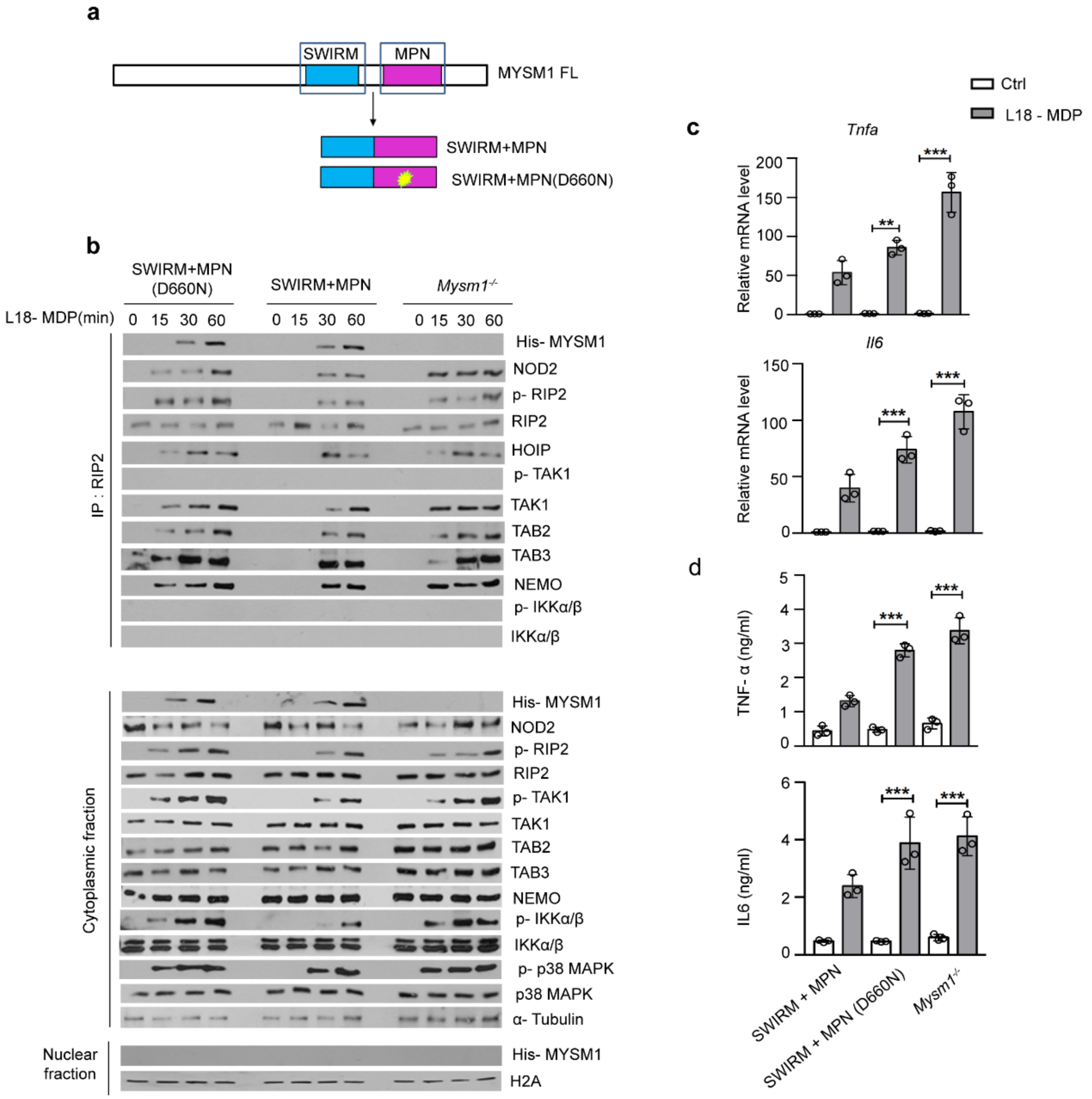

Supplementary Figure 7. Related to Figure 6. DUB activity of MYSM1 is required for attenuation but not interaction with RIP2. (**a**) Schematics of full length (FL) MYSM1 or a fusion construct of SWIRM and MPN: the active SWIRM+MPN or the inactive SWIRM+MPN (D660N). (**b-c**), *Mysm1*$^{-/-}$ BMDMs complemented with SWIRM+MPN or SWIRM+MPN (D660N) were stimulated with L18-MDP for indicated duration. RIP2 immunoprecipitates as well as corresponding cytoplasmic or nuclear fractions (**b**) were immunoblotted for indicated signalling molecules. Data representative of three independent experiments. (**c**, **d**) *Mysm1*$^{-/-}$ BMDMs or *Mysm1*$^{-/-}$ BMDMs complemented with indicated constructs were stimulated with L18-MDP and were then analyzed for *Tnfa* and *Il6* transcripts 6 h later (**c**) or for secreted TNF-α and IL-6 after 12 h (**d**). Results in **c-d** are from three independent experiments. Data are shown as mean ± s.e.m., (n=3). ***$p < 0.001$ determined by one-way ANOVA followed by Bonferroni's post-test depicts statistical significance relative to Ctrl.

Figure 1a

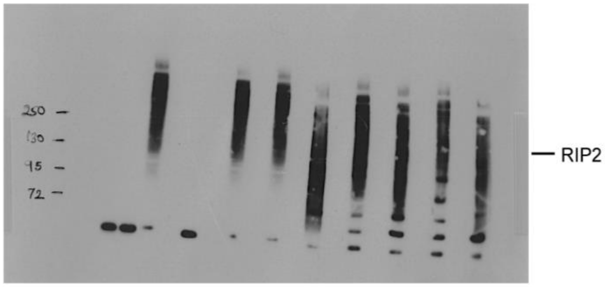

Figure 1c

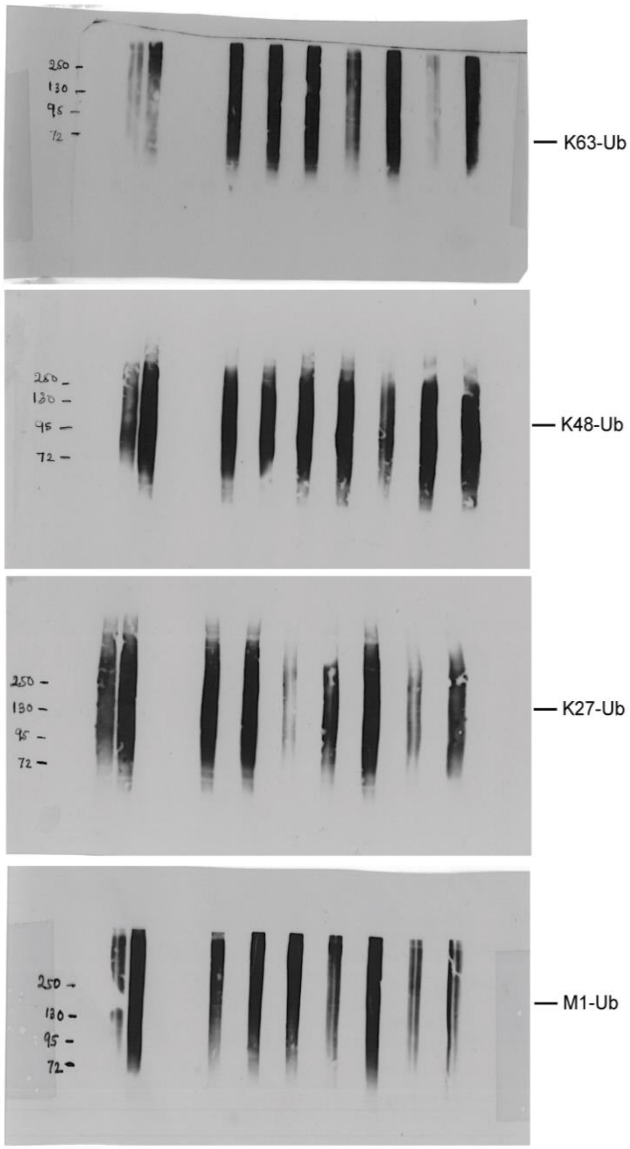

Supplementary Figure 8. Uncropped Immune blot for Figure 1a and 1c

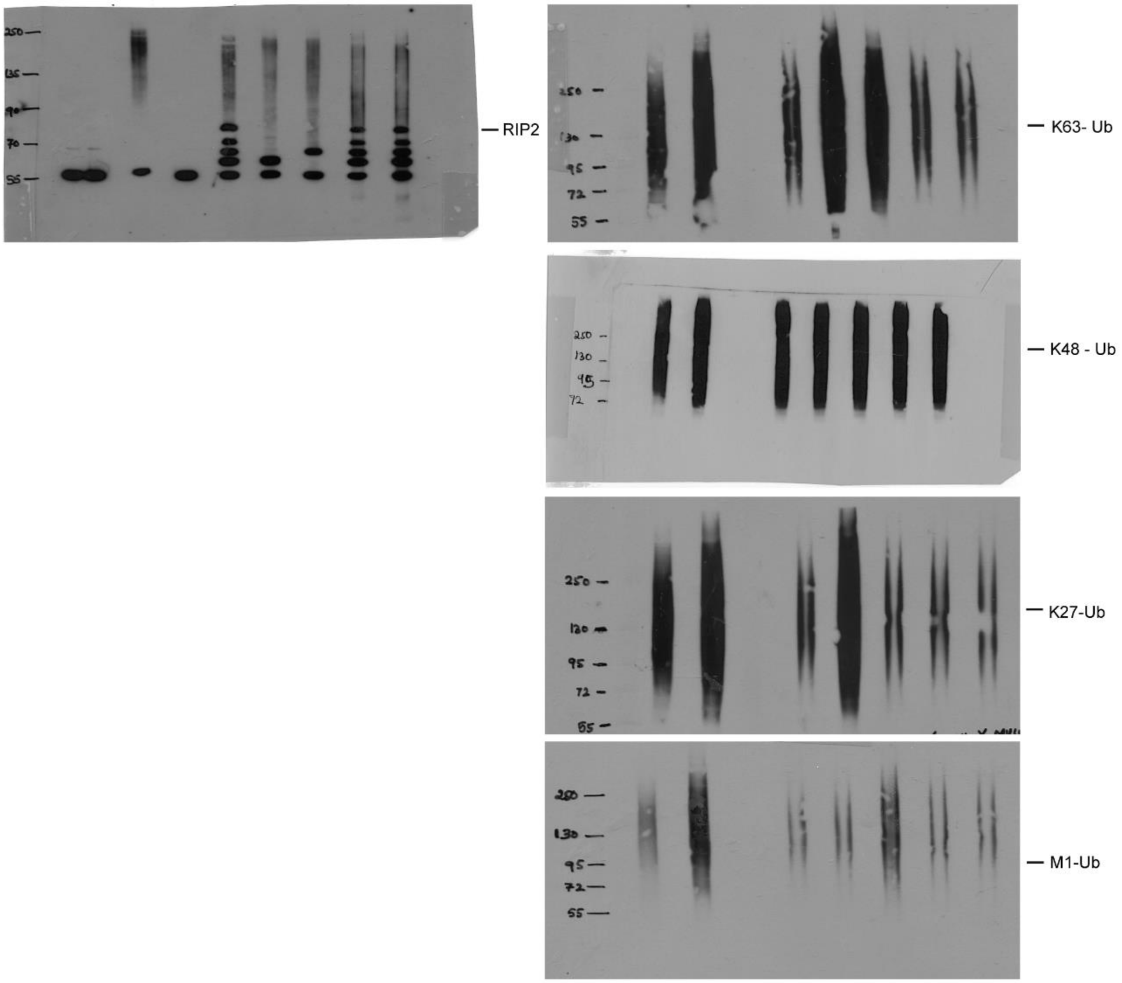

Supplementary Figure 9. Uncropped Immune blot for Figure 5b

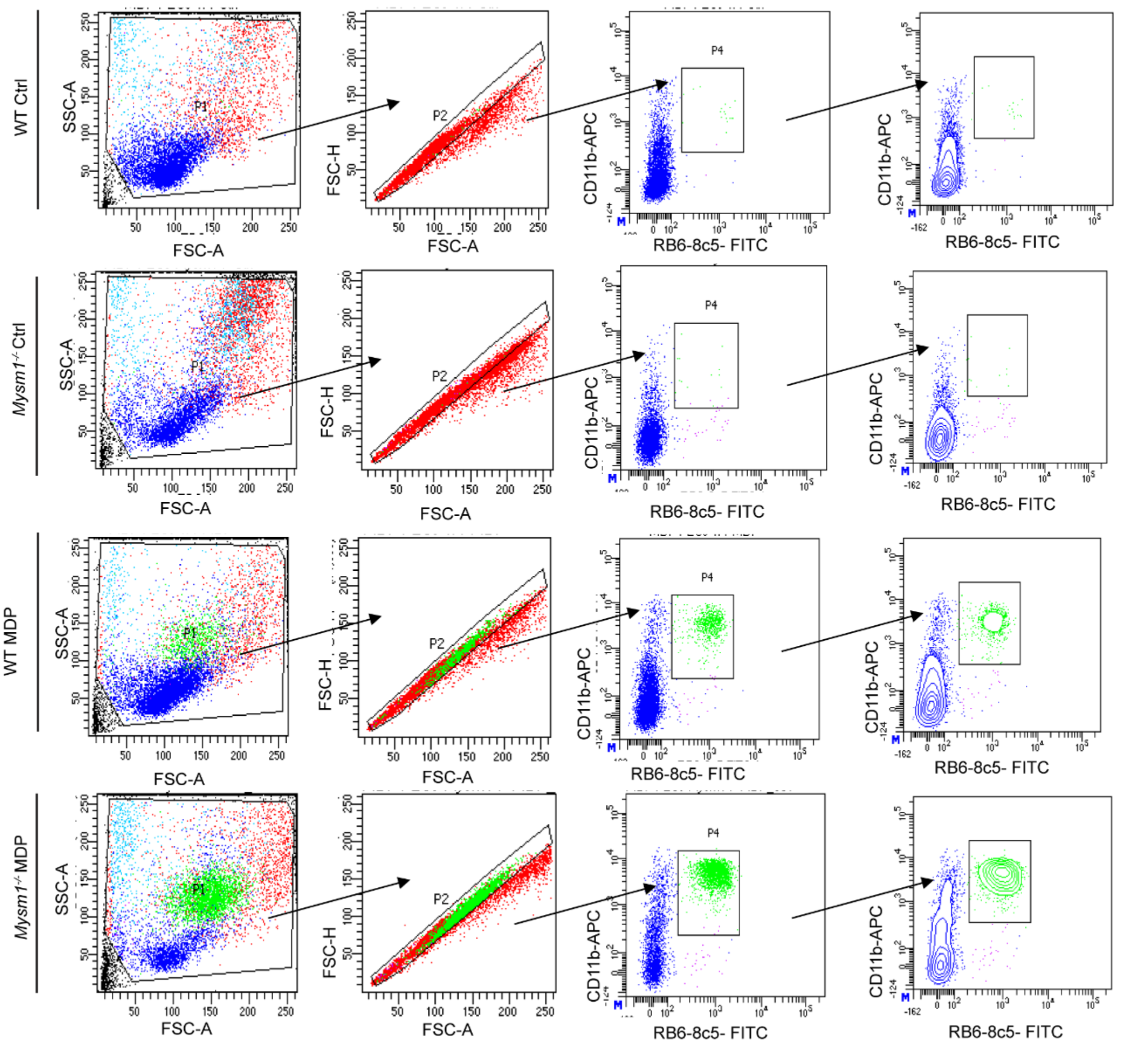

Supplementary Figure 10. Related to Figure 7. Gating strategies for the FACs data
