# Supplementary material for: The deubiquitinase MYSM1 dampens NOD2-mediated inflammation and tissue damage by inactivating the RIP2 complex
Source: Nat Commun. 2018 Nov 7;9:4654. doi: 10.1038/s41467-018-07016-0 (PMC6220254; doi:10.1038/s41467-018-07016-0)
Supplement: Supplementary file 3 — Reporting summary [file 41467_2018_7016_MOESM3_ESM.pdf]

nature research

Corresponding author(s): Gekara NO

Reporting Summary

Nature Research wishes to improve the reproducibility of the work that we publish. This form provides structure for consistency and transparency in reporting. For further information on Nature Research policies, see Authors & Referees and the Editorial Policy Checklist.

Statistical parameters

When statistical analyses are reported, confirm that the following items are present in the relevant location (e.g. figure legend, table legend, main text, or Methods section).

n/a	Confirmed	
☐	☒	The exact sample size (n) for each experimental group/condition, given as a discrete number and unit of measurement
☐	☒	An indication of whether measurements were taken from distinct samples or whether the same sample was measured repeatedly
☐	☒	The statistical test(s) used AND whether they are one- or two-sided<br>*Only common tests should be described solely by name; describe more complex techniques in the Methods section.*
☒	☐	A description of all covariates tested
☐	☒	A description of any assumptions or corrections, such as tests of normality and adjustment for multiple comparisons
☐	☒	A full description of the statistics including central tendency (e.g. means) or other basic estimates (e.g. regression coefficient) AND variation (e.g. standard deviation) or associated estimates of uncertainty (e.g. confidence intervals)
☐	☒	For null hypothesis testing, the test statistic (e.g. F, t, r) with confidence intervals, effect sizes, degrees of freedom and P value noted<br>*Give P values as exact values whenever suitable.*
☒	☐	For Bayesian analysis, information on the choice of priors and Markov chain Monte Carlo settings
☒	☐	For hierarchical and complex designs, identification of the appropriate level for tests and full reporting of outcomes
☒	☐	Estimates of effect sizes (e.g. Cohen's d, Pearson's r), indicating how they were calculated
☐	☒	Clearly defined error bars<br>*State explicitly what error bars represent (e.g. SD, SE, CI)*

Our web collection on statistics for biologists may be useful.

Software and code

Policy information about availability of computer code

Data collection	BD FACSDIVA software was used
Data analysis	Graphpad Prism software (version 7.04) was used for statistical analysis of cytokine/ ALT measurements. Immunoflurescence images were collected using Nikon's NIS Elements software (Nikon, version 3.2). Prism software (Graphpad) was used to plot bar graphs. BD FACSDIVA was used for FACs analysis. Adobe Photoshop (Adobe) was used to generate figures in the manuscript.

For manuscripts utilizing custom algorithms or software that are central to the research but not yet described in published literature, software must be made available to editors/reviewers upon request. We strongly encourage code deposition in a community repository (e.g. GitHub). See the Nature Research guidelines for submitting code & software for further information.

Data

Policy information about availability of data

All manuscripts must include a data availability statement. This statement should provide the following information, where applicable:

- Accession codes, unique identifiers, or web links for publicly available datasets
- A list of figures that have associated raw data
- A description of any restrictions on data availability

The raw data for all the figures are available.

Field-specific reporting

Please select the best fit for your research. If you are not sure, read the appropriate sections before making your selection.

☒ Life sciences ☐ Behavioural & social sciences ☐ Ecological, evolutionary & environmental sciences

For a reference copy of the document with all sections, see nature.com/authors/policies/ReportingSummary-flat.pdf

Life sciences study design

All studies must disclose on these points even when the disclosure is negative.

Sample size	For measurement of cytokine and ALT levels in serum samples, 4 mice per group/ genotype were used. For in vitro experiments, three biological replicates per condition or treatment group were collected for analysis.The statistics are described in Figure legends.
Data exclusions	No data were excluded.
Replication	All the experiments were performed at least thrice and data pooled from all experiments. All the data was reliably reproducible. The exact number of independent experiments conducted for each expeirments are described in Figure legends.
Randomization	Mice were allocated into groups according to their genotypes. Further they were sex and age matched.
Blinding	No blinding was used.

Reporting for specific materials, systems and methods

Materials & experimental systems

n/a	Involved in the study	
☒	☐	Unique biological materials
☐	☒	Antibodies
☐	☒	Eukaryotic cell lines
☒	☐	Palaeontology
☐	☒	Animals and other organisms
☒	☐	Human research participants

Methods

n/a	Involved in the study	
☒	☐	ChIP-seq
☐	☒	Flow cytometry
☒	☐	MRI-based neuroimaging

Antibodies

Antibodies used	Primary antibodies used in the study were as follows : Anti-p-RIP2 (#4364),anti-RIP2 (#4142),anti-p-TAK1(#9339), anti-TAK1 (#4505), anti-p-IKKα/β (#2078), anti-IKKα/β (ab178870), anti-p-p38MAPK (#4631), anti-p38 MAPK (#9212), anti-xIAP (#2042), anti-H2A (#12349), anti-K63 (#5621) and anti-K48 (#8081) were from Cell signaling technology. Anti-MYSM1 (ab193081), anti-HOIP (ab85294) and anti-K27 (ab181537) were from Abcam. Anti-TAB2 (MA514818) was from Thermoscientific. Anti-NEMO (559675) was from BD Biosciences. Anti-TAB3 (sc-46550), anti-NOD2 (sc-30199), HRP-conjugated goat anti-mouse IgG (sc-2005) , goat anti-rabbit IgG (sc-2004) and donkey anti-goat IgG (sc-2020) were from Santacruz biotechnology.
Validation	All commercial antibodies were tested by the supplier and further validated by western blotting .

Eukaryotic cell lines

Policy information about cell lines

Cell line source(s)	The L292 cell line was used as a source of macrophage colony stimulating factor (MCSF) used for differentiation of bone marrow derived macrophages. Each experiment was conducted using macrophage freshly differentiated from mouse bone marrow progenitors.
Authentication	Not applicable
Mycoplasma contamination	The L292 cell line was tested and confirmed to be mycoplasma free.

Commonly misidentified lines (See ICLAC register)	Not applicable

Animals and other organisms

Policy information about studies involving animals; ARRIVE guidelines recommended for reporting animal research

Laboratory animals	Experiments were conducted using wild type, Mysm1-/- and Rip2-/- mice on C57/BL6 background. Both male and female mice, 8-12 week of age were used. BMDMs were generated by differentiating mouse bone marrow progenitors.
Wild animals	Not used
Field-collected samples	Not applicable

Flow Cytometry

Plots

Confirm that:

☒ The axis labels state the marker and fluorochrome used (e.g. CD4-FITC).

☒ The axis scales are clearly visible. Include numbers along axes only for bottom left plot of group (a 'group' is an analysis of identical markers).

☒ All plots are contour plots with outliers or pseudocolor plots.

☒ A numerical value for number of cells or percentage (with statistics) is provided.

Methodology

Sample preparation	Spleens and PECs were minced between the 3 ml syringe plunger and fine mesh in FACS buffer (PBS with 2%FBS) to obtain single-cell suspensions . After ACK (Ammonium Chloride) lysis of RBCs, cells were washed once and were incubated with antibodies for 45/60 min on ice.<br>Samples have been stained in round bottom 96 well plate in 20-30ul of antibody mix.
Instrument	LSRII (BD Biosciences)
Software	BD FACSDIVA was used to collect and analyze flow cytometry data.
Cell population abundance	> 10,000 cells were analysed
Gating strategy	FSC/SSC gating to exclude debris. No other gating was applied.

☒ Tick this box to confirm that a figure exemplifying the gating strategy is provided in the Supplementary Information.
